# Mitochondrial genome characterization of a *Reticulinasus* sp. (Argasidae: Ornithodorinae) parasitizing bats in Thailand

**DOI:** 10.1186/s13071-025-06697-z

**Published:** 2025-02-13

**Authors:** Siwaporn Tuangpermsub, Apinya Arnuphapprasert, Elizabeth Riana, Thongchai Ngamprasertwong, Morakot Kaewthamasorn

**Affiliations:** 1 Center of Excellence in Veterinary Parasitology, Department of Pathology, Faculty of Veterinary Science, Chulalongkorn University, Bangkok, 10330 Thailand; 2 Veterinary Pathobiology Graduate Program, Faculty of Veterinary Science, Chulalongkorn University, Bangkok, 10330 Thailand; 3 Faculty of Veterinary Science, Rajamangala University of Technology Srivijaya, Nakhon Si Thammarat, 80240 Thailand; 4 The International Graduate Program of Veterinary Science and Technology (VST), Faculty of Veterinary Science, Chulalongkorn University, Bangkok, 10330 Thailand; 5 Department of Biology, Faculty of Science, Chulalongkorn University, Bangkok, 10330 Thailand

**Keywords:** Bat, Tick, Mitochondrion genome, *Reticulinasus* sp., Thailand

## Abstract

**Background:**

Second only to mosquitoes, ticks (Acari: Ixodida) are significant blood-feeding ectoparasites and vectors of numerous pathogens affecting both animals and humans. Despite bats serving as hosts to various tick species, they remain relatively understudied due to their nocturnal behavior and laborious capture procedures. Soft ticks in particular display diverse ecological behaviors, inhabiting bat roosts, caves, and occasionally human dwellings. This overlap in habitats suggests soft ticks may play a critical role as vectors of zoonotic pathogens. In Southeast Asia, research on soft ticks has primarily focused on island nations, with limited studies on bat-associated ticks in Thailand. This study aimed to examine the identity and distribution of bat ticks in Thailand.

**Methods:**

Bats were captured across ten provinces in Thailand between 2018 and 2023. Ticks were removed from the bats’ skin and identified through morphological examination using a stereomicroscope, with molecular confirmation. Scanning electron micrographs were recorded. Prevalence, mean abundance, and mean intensity of tick infestations were calculated. The mitochondrial genomes of the ticks were sequenced, annotated, and subjected to phylogenetic analysis.

**Results:**

A total of 1031 bats, representing 7 families, 11 genera, and 28 species, were captured. Tick infestations were found in 34 bats (3.30%), specifically in two species: *Craseonycteris thonglongyai* (33/139, 23.74%) and *Eonycteris spelaea* (1/2, 50%). All ticks were in the larval stage. Basic local alignment search tool for nucleotide (BLASTN) searches using *16S* rRNA (425 bp) and *COI* (825 bp) sequences, along with Barcode of Life Database (BOLD) database queries, revealed the highest similarity to tick in the genus *Reticulinasus* found on bats in Zambia. The mitochondrial genomes of ticks collected from *C. thonglongyai* and *E. spelaea* were 14,433 bp and 14,439 bp in length, respectively, and contained 13 protein-coding genes, 22 tRNA genes, and 2 rRNA genes. Phylogenetic analysis placed these ticks within the *Reticulinasus* clade, with strong support indicated by high bootstrap values.

**Conclusions:**

This study identified *Reticulinasus* sp. infestations on *C. thonglongyai* and *E. spelaea* bats, marking the first report of soft ticks in bats from Thailand, with potential implications for zoonotic disease transmission.

**Graphical Abstract:**

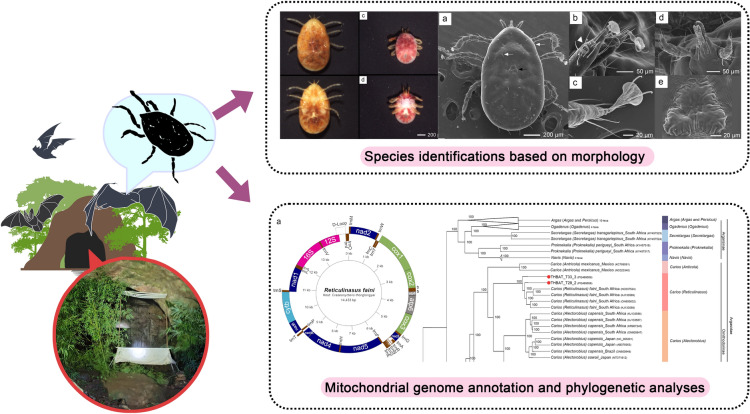

**Supplementary Information:**

The online version contains supplementary material available at 10.1186/s13071-025-06697-z.

## Background

Ticks (Acari: Ixodida) are significant blood-feeding ectoparasites and important vectors of several pathogens affecting both animals and humans, ranking second only to mosquitoes in terms of human disease transmission. However, ticks are the primary source of pathogen transmission to animals. Ticks comprise at least 960 species worldwide, belonging to three major families: Ixodidae (hard ticks), Argasidae (soft ticks), and Nuttalliellidae [1–3]. According to the current list, there are 700 extant species of ixodids and 193 species of argasids [4]. Several soft tick species are considered cryptic, indicating that the full diversity of soft ticks has not yet been fully described. Historically, no consensus classification scheme for soft ticks has existed, as most systems relied solely on unique morphological characters (synapomorphies) [5]. Consequently, molecular approaches provide researchers with greater confidence in identifying morphotaxonomic groups. For instance, Muñoz-Leal et al. [6] and Mans et al. [5, 7] utilized mitochondrial and nuclear genome studies to support the reclassification of soft tick taxonomy. Notably, more than 30 new argasid species have been described, revised, or had nucleotide data, including mitochondrial genome sequences, deposited in public databases since the compilation of this list [1, 5–7]. This indicates the continuous expansion and development in the field of soft tick taxonomy. Argasid classification, however, remains controversial since it does not reflect evolutionary relationships and results in paraphyly for the main genera of soft ticks (Argasidae), namely *Argas* and *Ornithodoros*.

Bats are reservoirs of several infectious disease agents and host a wide diversity of hematophagous arthropods, including ticks. Soft ticks exhibit diverse behaviors, residing not only in nests, burrows, and crevices in caves, but also in human dwellings. In the adult stage, they take a few minutes to a few hours to feed on the blood of a host before taking off and living in the environment. These behaviors indicate that soft ticks, which share habitats with humans and other animals, play a potentially crucial role as vectors for several pathogens, including those with zoonotic potential. For instance, ticks in the genera *Ornithodoros* and *Argas* are primarily responsible for transmitting *Borrelia* sp., which can cause relapsing fever in humans [8, 9]. *Babesia vesperuginis* has been detected in *Argas vespertilionis* in Hungary and China [10]. *Rickettsia* spp. have been found in several genera of soft ticks, including *Argas, Ornithodoros, Reticulinasus*, and *Carios,* in multiple regions [11–15]. Additionally, certain species of soft ticks have a close association with bats. Numerous reports worldwide have documented the infestation of bats by various species of soft ticks. In Brazil alone, at least nine species of soft ticks parasitize bats [16], while in the Western Palearctic, at least five species have been found on bats [17].

In Southeast Asia, several groups of researchers have investigated soft ticks and their distribution in the island nations of the region [18]. However, there have been limited studies on bat ticks and their associated bat species in Thailand despite the fact that Thailand is home to at least 148 bat species, accounting for nearly 10% of bat species documented worldwide [19]. Therefore, this study aimed to investigate the distribution and characterize the species of bat-associated ticks in Thailand.

## Methods

### Description of sampling sites, bat capture, and tick collection methods

**Fig. 1:**
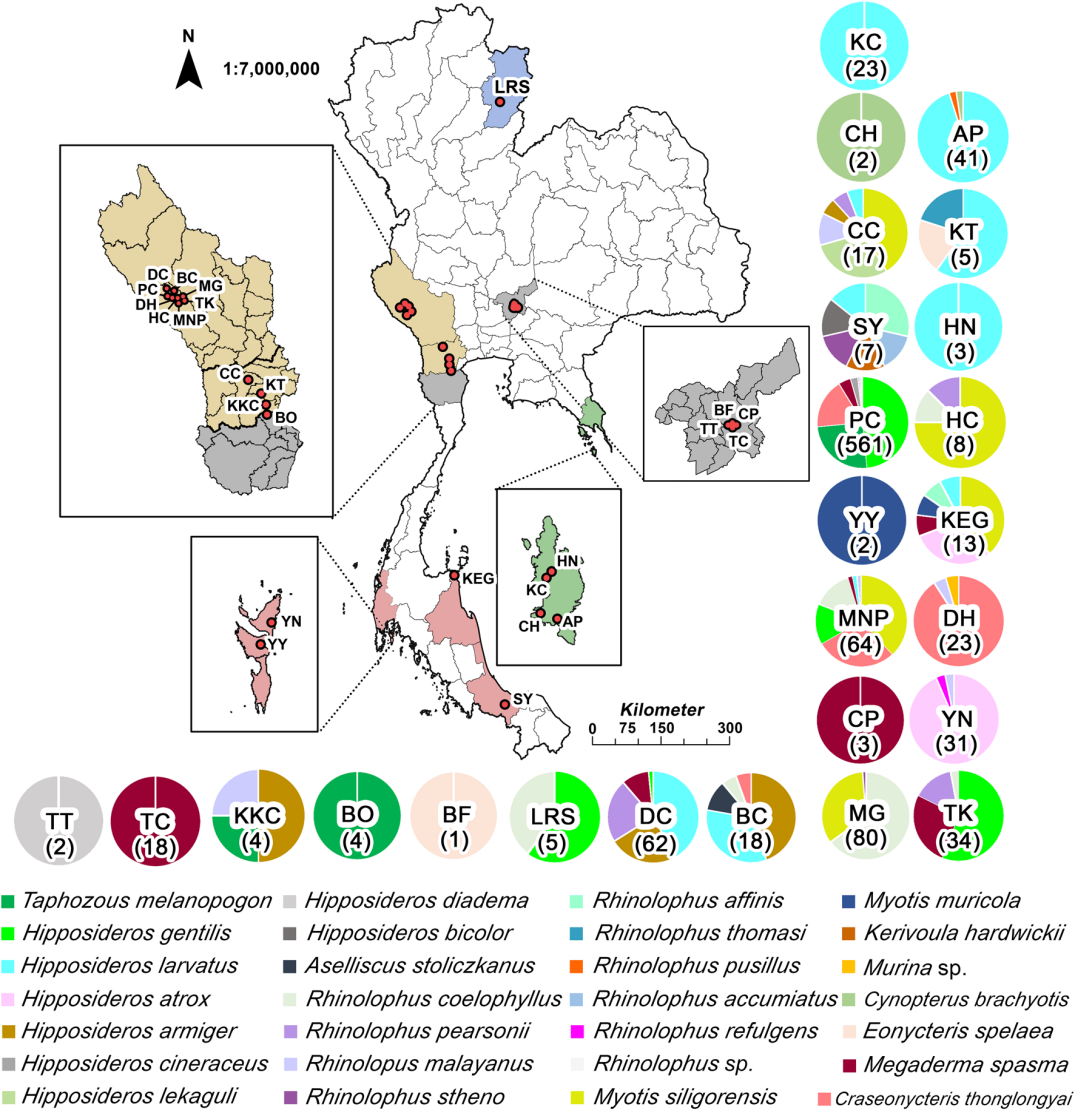
Locations and numbers of bats captured across various sites in Thailand (detailed in Table S3). The map depicts bat capture locations, with the number of bats captured at each site indicated in parentheses. The sites are as follows: Lainan Research Station (LRS), Daowadung Cave (DC), Bat Cave (BC), Ma Gleua Cave (MG), Dhepnimitra Cave (DH), Ta-Klor Cave (TK), Ma Now Phee (MNP), Phra Cave (PC), Hintok Cutting (HC), Chomphon Cave (CC), Kunchorn Temple (KT), Khun Kunchon Cave (KKC), Bo Cave (BO), Banana Farm (BF), Teak Tree Forest (TT), Tiger Cave (TC), Champa Cave (CP), Khanom Electricity Generating Co. Ltd. (KEG), Saba Yoi (SY), Yao Noi Island (YN), Yao Yai Island (YY), Huang Nam Khiao Waterfall (HN), Cham’s House (CH), Khlong Chao Waterfall (KC), and Ao Phrao Temple (AP). Bat species captured at each site are represented using color codes

This study was undertaken as part of a bat pathogen survey, which was previously described by Arnuphapprasert et al. [20], Poofery et al. [21], and Riana et al. [22]. Bats were captured using mist nets, tunnel nets, and harp traps in front of caves or along flight paths. Bats inside the caves were caught using hand nets. The trapping activities were carried out from February 2018 to February 2023, covering ten provinces across five geographical regions in Thailand (Fig. 1). The sampling sites included Saraburi (2018–2020) in the central region; Trat (2019) in the eastern region; Nan (collected in 2020) in the northern region; Kanchanaburi (2018–2023), Ratchaburi (2019–2020), and Phetchaburi (2020) in the western region; and Songkhla (2018), Krabi (2021), Phang-nga (2021), and Nakhon Si Thammarat (2022) in the southern region. Upon capture, each bat was carefully placed in an individual cloth bag. The identification of bat species relied primarily on morphological keys, and when the morphology was ambiguous, they were confirmed using echolocation calls. Sex, reproductive status, and capturing location data were recorded. Before their release alive, the bats were examined for ectoparasites, and ticks were collected using fine forceps and preserved in sterile microcentrifuge tubes containing 70% or absolute ethanol. All specimens were kept in a box with ice packs and transported to the laboratory at the Faculty of Veterinary Science, Chulalongkorn University. Subsequently, they were stored at −40 °C until further processing.

### Tick identifications

Bat ticks were morphologically examined using an Olympus SZ61 Stereozoom Microscope (Olympus Co., Ltd., Tokyo, Japan), a Nikon Upright Microscope ECLIPSE Si RS (Nikon Co., Ltd., Tokyo, Japan) equipped with a Lanoptik HD1210W-N Microscope camera (Lanoptik Technologies Ltd., Guangzhou, China), and a JEOL JSM-IT300 Scanning Electron Microscope (SEM) (JEOL Ltd., Tokyo, Japan). Identification keys [23–27] were used to determine the stage and species of the ticks. For examination under the light microscope, ticks were mounted onto glass slides following the clearing and staining protocol of Paulraj et al. [28]. Subsequently, tick DNA samples were extracted individually using a NucleoSpin® Tissue extraction kit (Macherey–Nagel, Germany) according to the manufacturer’s instructions with slight modifications on the final elution step. The final elution volume was 25 µL to concentrate the amount of recovered nucleic acids. The tick 16S mitochondrion gene fragment was the first target to amplify using conventional polymerase chain reaction (PCR) with primers previously described by Norris et al. [29] and Mangold et al. [30]. The primer sequences are provided in Supplementary Table S1. These primers were utilized to amplify a wide range of tick species, producing a fragment corresponding to the *Drosophila yakuba* 16S rDNA sequence between positions 12,887 and 13,431 [31]. In the initial PCR attempt, no positive control was included due to the unavailability of a known DNA sample. Sterile distilled water was used as a negative control to check the absence of contamination. Subsequently, the purified PCR product of the tick 16S mitochondrial gene fragment from the first attempt, which had been confirmed both morphologically and through sequencing as *Reticulinasus* sp., was utilized as the positive control for subsequent PCR reactions. Each PCR reaction comprised a 12.5 µL master mix, consisting of 0.25 µL of KOD Fx Neo polymerase (Toyobo, Japan), 6.25 µL of 2X KOD Fx Neo Buffer, 2.5 µL of dNTPs (0.4 mM each), 1.75 µL of sterile distilled water, 0.375 µL of each primer (final concentration = 0.3 µM), and 1 µL of genomic DNA. The amplification was carried out using a MiniAmp™ Thermal cycler (Thermo Fisher Scientific, USA). A total of 5 microliters of PCR product, along with loading dye, were loaded onto a 1.5% agarose gel. Electrophoresis was performed at 100 V and 400 mA for 40 min, followed by a visual examination of the results under UV light. Subsequently, the PCR-positive products were purified using NucleoSpin® Gel and a PCR Clean-up Kit (Macherey–Nagel, Germany) following the steps in the instruction manual. Subsequently, the samples were sent to the Sanger DNA sequencing service (U2Bio Co., Ltd, South Korea) in both directions. Chromatograms obtained from the sequencing of the tick 16S mitochondrial gene fragment were examined, manually edited, and assembled using BioEdit software (version 7.0.5.3) [32]. Basic local alignment search tool for nucleotide (BLASTN) searches were carried out through the GenBank database (https://blast.ncbi.nlm.nih.gov) to provide initial identification of the most closely related tick genus or species.

### PCR amplifications and sequencing of whole mitochondrial genes

**Fig. 2:**
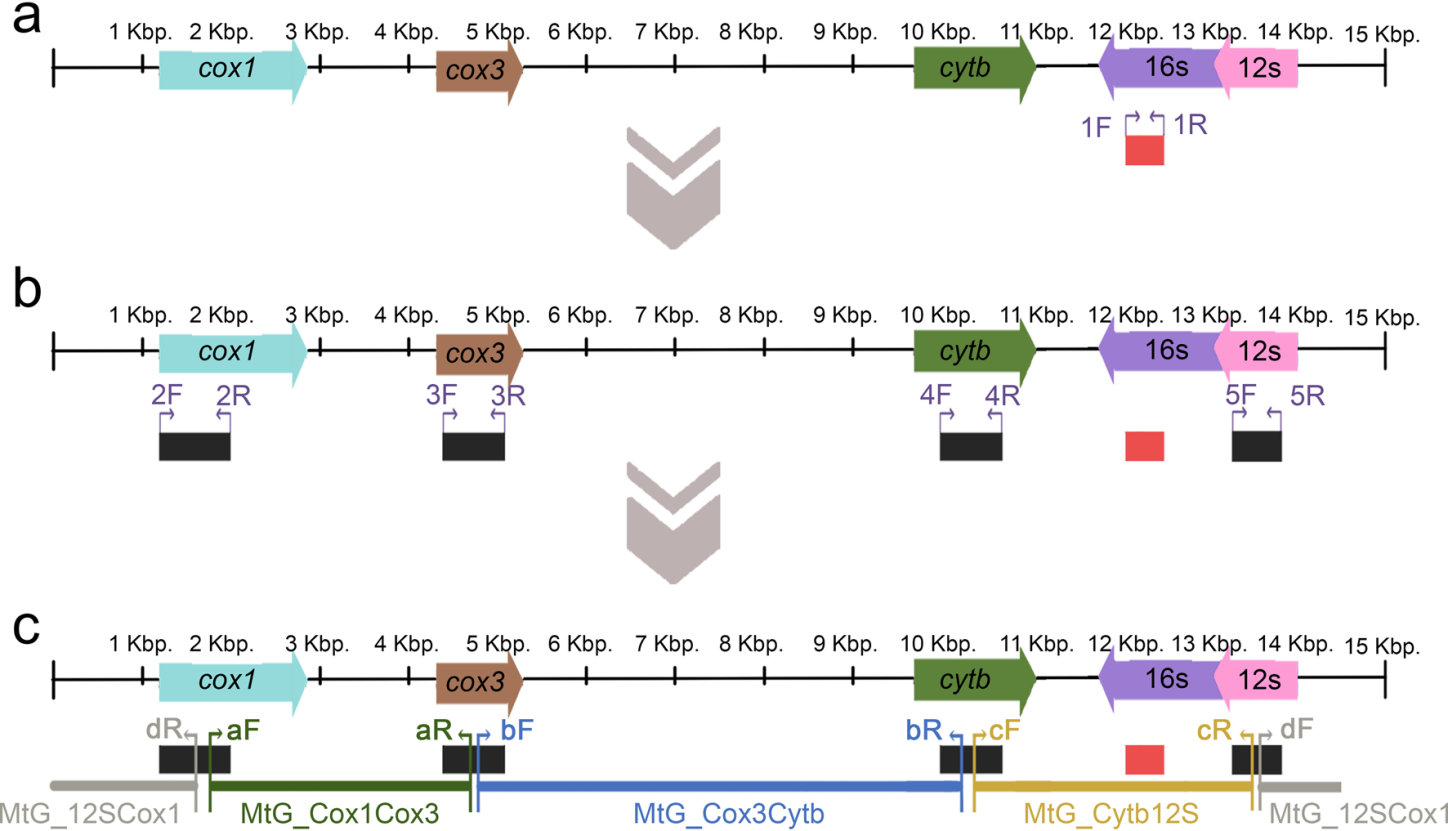
**a** Linear representation of the mitochondrial genome of soft ticks for clarity, adapted from the depiction by Mans et al. [5]. Arrows indicate the gene arrangement selected for primer design in this study. Primer sets 1F and 1R, identified as universal primers targeting the 16S rRNA gene for tick species identification, are designed to amplify a 460 bp gene fragment. The primer sequences for this 16S rRNA gene fragment are provided in Supplementary Table S1. **b** Following preliminary species identification using the 16S rRNA sequence, four primers (2F, 2R, 3F, 3R, 4F, 4R, 5F, and 5R) listed in Table S1 were used to amplify four short mitochondrial gene fragments: *cox1* (845 bp), *cox3* (690 bp), *cytb* (800 bp), and *12S* (530 bp). **c** These short mitochondrial gene sequences were subsequently used to design four additional primer sets (aF, aR, bF, bR, cF, cR, dF, and dR) for the amplification of long mitochondrial gene fragments with lengths of 2925 bp, 5475 bp, 3130 bp, and 1280 bp, respectively. Details of the primer sequences and their target fragments are provided in Supplementary Table S2. In all primer names, “F” denotes the forward primer and “R” denotes the reverse primer for both short and long mitochondrial fragments. Once all amplicons were generated, the full mitochondrial genome sequence was assembled

The complete mitochondria genome sequences of ticks currently available in the public database were over 14,000 bp and in circular form [5]. In the initial step, four short DNA fragments were amplified and sequenced, using available mitochondrial genome sequences of ticks as templates for primer design. The initial BLASTN results revealed the highest similarity between the mitochondrial 16S rRNA fragment from the ticks in this study and *R. faini* (GenBank accession numbers LC190988 and LC634598), which guided the primer selection. Mitochondrial genomes of closely related tick species were also considered during primer design. Therefore, primer design in this study was informed by the alignment of mitochondrial genomes of ticks, using the following GenBank references: NC_037524, ON800832, ON800847, KJ133588 (for *R. faini*); AB075953 (for *Alectorobius capensis*); KJ133604, KJ133603 (for *Ornithodoros savignyi*); KX712088 (for *Nothoaspis amazoniensis*); and OM368318 (for *Carios vespertilionis*). To obtain a complete mitochondrial genome sequence of ticks in this study, two rounds of PCR amplification were performed: first, the amplification of four short fragments, followed by the amplification of four long fragments (Fig. 2). In total, four primer sets were designed to amplify short fragments of the cytochrome c oxidase I (*cox1*), cytochrome c oxidase III (*cox3*), cytochrome b (*cytb*), and 12S ribosomal RNA (12S rRNA) genes. The PCR products of these short fragments were then amplified, purified, and sent for Sanger DNA sequencing (U2Bio Co., Ltd, Korea). All primers and PCR protocols used in this study are provided in Supplementary Table S1. After obtaining the four short mitochondrial gene fragments, these were used as templates to design a second set of primers, which were employed to amplify longer fragments, thereby completing the sequencing of the remaining gaps in the mitochondrial genome of the bat-associated tick. The primers and PCR protocols employed in this study can be found in Supplementary Table S2. Each PCR reaction for long fragment comprised a 12.5 μL master mix, consisting of 0.125 μL of Takara LA Taq polymerase (Takara Bio, USA), 1.25 μL LA PCR Buffer II, 2 μL of dNTPs (0.4 mM each), 5.735 μL of sterile distilled water, 0.5 μL of each primer (final concentration = 0.3 μM), 1.25 MgCl_2_ and 1.5 μL of genomic DNA. A MiniAmp™ Thermal Cycler (Thermo Fisher Scientific, USA) was used to amplify long fragments. A total of 5 microliters of PCR product, along with loading dye, were loaded onto 0.8% agarose gel. Electrophoresis was performed at 100 V and 400 mA for 45 min, followed by visual examination of the results under UV light. According to the instruction manual, the PCR-positive products were purified using a NucleoSpin® gel purification kit (Macherey–Nagel, Germany) and subsequently sequenced by an NGS-based innovative BTSeq™ (Barcode-Tagged Sequencing) platform utilizing the MiSeq Reagent Kits v2 Illumina sequencer (Illumina, USA), which was performed by U2Bio Company Limited in South Korea.

### Nucleotide sequence processing, phylogenetic and statistical analyses

The sequence results were inspected and manually edited where necessary on the basis of chromatograms obtained from both directions and aligned using BioEdit v7.0.5.3 [32]. Afterward, all fragments were assembled using Uniprot UGENE [33] to create the completed mitochondrial genome sequence of the tick. For the protein-coding genes (PCGs), sequences were aligned and translated into amino sequences to verify there was no internal stop codon using Translator X [34]. For non-protein-coding genes, sequences were rechecked with reference data on the GenBank database. Sequences were selected to identify tick species using the BLASTN program on the NCBI website (https://blast.ncbi.nlm.nih.gov/Blast.cgi). In addition, mitochondrial *cox1* sequences were utilized to conduct a search in the Barcode of Life Data System (BOLD) (https://boldsystems.org) for further species identification. For annotated genes on mitochondria, MITOS WebServer [35] was used to locate the gene position, and they were manually checked and edited following the reference sequence of *R. faini* tick (accession no. ON800832). A neighbor-joining (NJ) tree was created using MEGA software, employing the Kimura two-parameter distance model [36] for all pairwise comparisons of 16S rRNA sequences. The 16S rRNA sequences of soft-tick species, available in the GenBank database, were utilized to assess intraspecific genetic distances. To construct the phylogenetic tree, the concatenated sequences of 13 PCGs and 2 ribosomal genes (13,445 bp) from two mitochondrion sequences in this study and 83 sequences retrieved from the GenBank database were selected (Supplementary Table S6). Both maximum likelihood (ML) and Bayesian inference (BI) methods were used to conduct the phylogenetic analysis. Two sequences of *Ixodes simplex* (KY457532 and NC062060) were used as the outgroup. All sequences were aligned using ClustalW via Job Dispatcher [37], and the ambiguous region was removed. Maximum likelihood analysis was performed by IQ-TREE multicore version 1.6.12 [38] with optimal model GTR + F + I + G4, which was calculated by ModelFinder. Bayesian inference was performed by MrBayes on XSEDE (3.2.7a) access through the CIPRES gateway (https://www.phylo.org/). Posterior probabilities (pp) were calculated using Markov chain Monte Carlo (MCMC) sampling, which involved running two parallel chains for 100 million generations. The most appropriate substitution model was selected using Markov chain sampling over the GTR model space. The phylogram was drawn using the FigTree v.1.4 program (http://tree.bio.ed.ac.uk/software/figtree) and edited with Adobe Photoshop®. To determine the prevalence, mean abundance, and mean intensity of tick infestation, QPweb was used [39].

## Results

### Bats captured and prevalence of tick infestation.

This study was conducted across 25 sampling locations in 10 provinces of Thailand (Fig. 1). Over a 5-year period, from February 2018 to February 2023, a total of 1031 bats were captured successfully, representing 7 families, 11 genera, and 28 species. The annual distribution of captured bats is as follows: 41 in 2018, 234 in 2019, 327 in 2020, 130 in 2021, 239 in 2022, and 60 in 2023. The majority of bats were captured in Kanchanaburi, a province located in western Thailand, with 850 bats captured across 8 locations. Trat Province recorded the second-highest number of captures, with 69 bats from 4 locations. A detailed summary of all bats captured in this study can be found in Supplementary Table S3. Out of 1031 bats, 34 individuals (3.30%) were found to be infested with soft ticks. Notably, soft ticks were detected on only two bat species: *C. thonglongyai* (33/139, 23.74%) and *E. spelaea* (1/2, 50%). Focusing on tick-infested bats at each location, the highest prevalence, mean abundance, and mean intensity were observed in *C. thonglongyai* from Manow Phee Cave in Kanchanaburi Province (mean abundance: 2.22 ± 5.16; mean intensity: 4.00 ± 6.52; *P* = 0.43; Table 1). Most ticks were collected during the dry season (mid-October to early May), although some samples were also collected during the wet season.

**Table 1:** Frequency of tick infestation, mean intensity, and mean abundance of ticks in two bat species across different locations

Bat species	Sampling location (Province)	Number of bat capture	Number of ticks infested bat	Number of ticks	Prevalence %	Mean abundant (95% CI)	Mean intensity (95% CI)
*Eonycteris spelaea*	Banana farm (Saraburi)	1	1	4	100	4	4
	Kunchorn temple (Ratchaburi)	1	0	0	–	–	–
*Craseonycteris thonglongyai*	Phra cave (Kanchanaburi)	99	16	31	16.2	0.31 (± 0.90)	1.94 (± 1.39)
	Dhepnimitra cave (Kanchanaburi)	21	7	21	33.3	1.00 (± 1.64)	3.00 (± 1.41)
	Manow Phee cave (Kanchanaburi)	18	10	40	55.6	2.22 (± 5.16)	4.00 (± 6.52)
	Bat cave (Kanchanaburi)	1	0	0	–	–	–
	Total	141	34	96			

### Species identifications based on morphology and nucleotide sequences

**Fig. 3:**
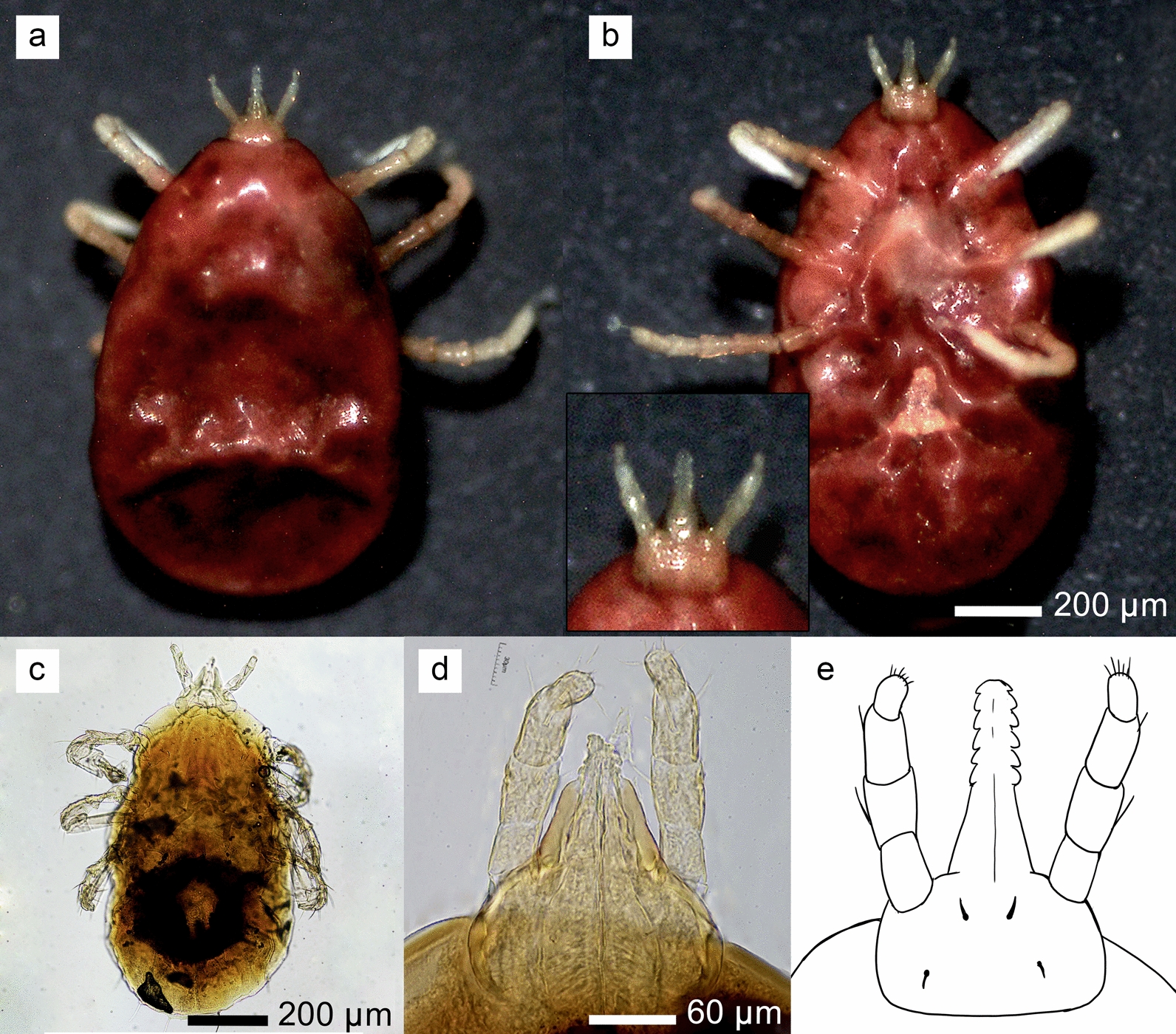
Morphological characteristics of soft tick larvae collected from bats, observed using stereomicroscopy and light microscopy: **a**, **b** Dorsal and ventral views of a larva collected from a *Craseonycteris thonglongyai* bat, observed under a stereomicroscope, with an inset highlighting the capitulum region; **c** whole body of a larva after slide mounting, observed under a light microscope; **d** detailed view of the basis capitulum from the slide-mounted larva, as observed under a light microscope; **e** an illustration depicting the structural features of the basis capitulum

**Fig. 4:**
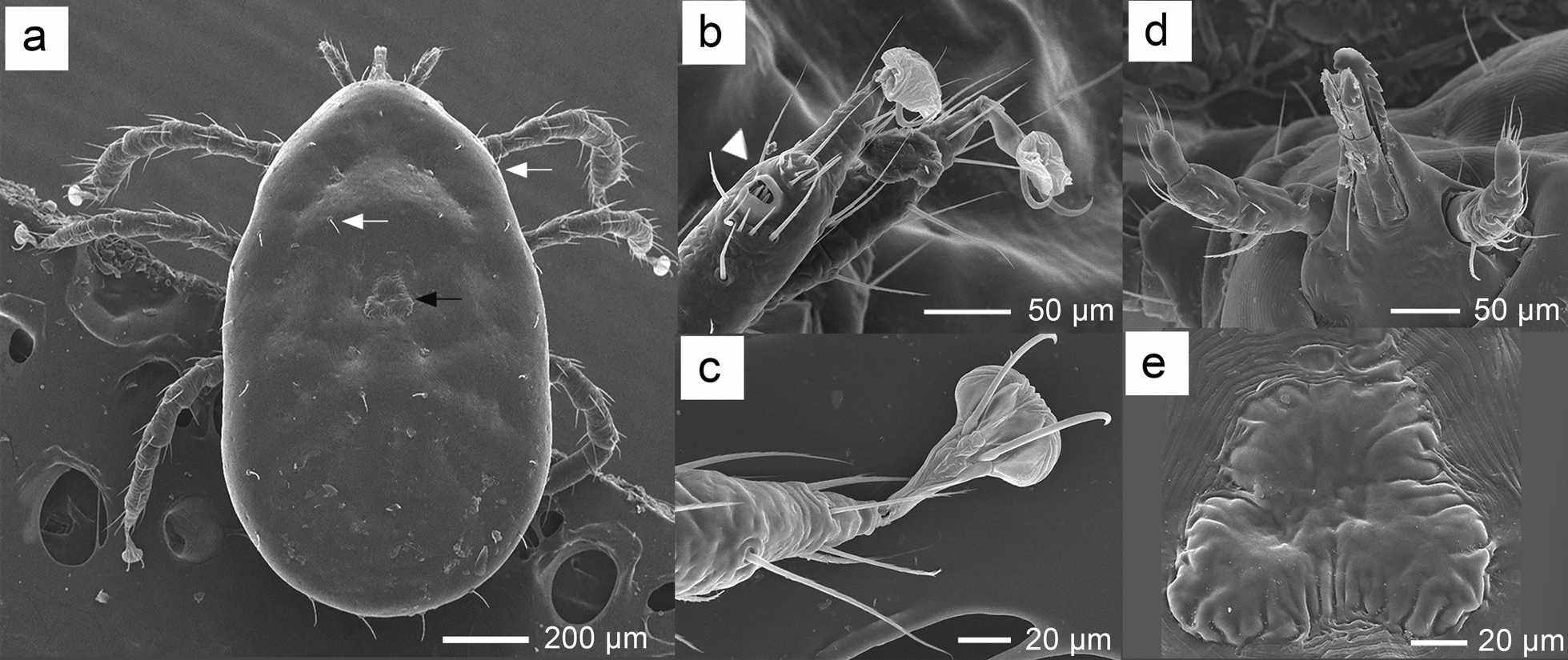
Morphological characteristics of the larval stage of *R. faini* as observed under scanning electron microscopy (SEM): **a** idiosoma in dorsal view; the white arrow indicates the dorsal lateral and central setae, while the black arrow points to the dorsal plate; **b** legs of the tick; white arrowhead highlights Haller’s organ located on tarsus I, showing a perforated capsule; **c** claws with pulvillus; **d** gnathosoma and hypostome in ventral view; **e** dorsal plate exhibiting a triangular shape

A total of 96 ticks collected from bat hosts were identified as larvae of soft ticks (Figs. 3 and 4). The larvae ranged in body size from 0.8 mm to 1.5 mm, with most specimens appearing engorged. Optical micrographs obtained via stereomicroscopy and light microscopy revealed that the hypostome had a rounded apex. Additionally, the hypostome of the larvae arose from a median truncated extension of the basis capitulum rather than directly from the basis capitulum. Further analysis with scanning electron microscopy (SEM) (Fig. 4) revealed a rounded, anterior triangular dorsal plate measuring 0.09–0.11 mm in length and 0.09–0.1 mm in width. In all specimens, the dorsolateral and central setae were needle-like, totaling 14 pairs: 12 dorsolateral pairs and 2 central pairs. Additionally, the Haller’s organ, located on tarsus I, exhibited a characteristic capsule-perforated structure. For a total of 13 tick specimens out of 96, which were randomly selected for sequencing of the mitochondrial 16S rRNA and *cox1* genes, the BLASTN analysis of the 16S rRNA sequences (425 bp) indicated the highest similarity to *R. faini*, a tick species collected from Zambia (GenBank accession numbers LC190988 and LC634598), with a percentage identity ranging from 98.59% to 99.06% (Table 2). Similarly, the *cox1* sequence analysis (825 bp) using the BOLD database indicated the closest match to *R. faini* from Zambia (BOLD IDs: ADC5885 and ADC5885), with percent similarity ranging from 94.26% to 96.09%.

**Table 2:** BLASTN search results for *16S rRNA* and BOLD search results for *cox1* of tick samples in this study

Sample name	Sampling location (Province)	Bat host species	BLASTN result of *16S rRNA* (429 bp)			BOLD results of *cox1* (825 bp)		
			Most closely related to tick species	Accession no.	% identity	Most closely related to tick species	BOLD ID	% identity
THBAT_T01_2	Manow Phee cave (Kanchanaburi)	*C. thonglongyai*	*R. faini*	LC190988	99.06	*R. faini*	BOLD: ADC5885	96.09
				LC634598	99.06		BOLD: ADR8307	94.99
THBAT_T01_3	Manow Phee cave (Kanchanaburi)	*C. thonglongyai*	*R. faini*	LC190988	99.06	*R. faini*	BOLD: ADC5885	95.85
				LC634598	99.06		BOLD: ADR8307	94.75
THBAT_T06_3	Manow Phee cave (Kanchanaburi)	*C. thonglongyai*	*R. faini*	LC190988	99.06	*R. faini*	BOLD: ADC5885	95.85
				LC634598	99.06		BOLD: ADR8307	94.75
THBAT_T11_1	Phra cave (Kanchanaburi)	*C. thonglongyai*	*R. faini*	LC190988	99.06	*R. faini*	BOLD: ADC5885	96.09
				LC634598	99.06		BOLD: ADR8307	94.99
THBAT_T19_1	Dhepnimitra Cave (Kanchanaburi)	*C. thonglongyai*	*R. faini*	LC190988	99.06	*R. faini*	BOLD: ADC5885	96.09
				LC634598	99.06		BOLD: ADR8307	94.99
THBAT_T23_2	Dhepnimitra Cave (Kanchanaburi)	*C. thonglongyai*	*R. faini*	LC190988	99.06	*R. faini*	BOLD: ADC5885	96.09
				LC634598	99.06		BOLD: ADR8307	94.99
THBAT_T27_1	Dhepnimitra Cave (Kanchanaburi)	*C. thonglongyai*	*R. faini*	LC190988	99.06	*R. faini*	BOLD: ADC5885	96.09
				LC634598	99.06		BOLD: ADR8307	94.99
THBAT_T28_2	Phra cave (Kanchanaburi)	*C. thonglongyai*	*R. faini*	LC190988	99.06	*R. faini*	BOLD: ADC5885	96.09
				LC634598	99.06		BOLD: ADR8307	94.99
THBAT_T29_2	Phra cave Kanchanaburi	*C. thonglongyai*	*R. faini*	LC190988	99.06	*R. faini*	BOLD: ADC5885	96.09
				LC634598	99.06		BOLD: ADR8307	94.99
THBAT_T31_1	Phra cave (Kanchanaburi)	*C. thonglongyai*	*R. faini*	LC190988	99.06	*R. faini*	BOLD: ADC5885	96.09
				LC634598	99.06		BOLD: ADR8307	94.99
THBAT_T32_1	Phra cave (Kanchanaburi)	*C. thonglongyai*	*R. faini*	LC190988	99.06	*R. faini*	BOLD: ADC5885	96.09
				LC634598	99.06		BOLD: ADR8307	94.99
THBAT_T33_3	Banana farm (Saraburi)	*E. spelaea*	*R. faini*	LC190988	98.59	*R. faini*	BOLD: ADC5885	94.26
				LC634598	98.59		BOLD: ADR8307	93.53
THBAT_T33_4	Banana farm (Saraburi)	*E. spelaea*	*R. faini*	LC190988	98.59	*R. faini*	BOLD: ADC5885	94.26
				LC634598	98.59		BOLD: ADR8307	93.53

### Sequence analysis, mitochondrial genome annotation, and phylogenetic analyses

The 13 16S rRNA sequences obtained in this study revealed only two distinct haplotypes. Additionally, four publicly available 16S rRNA sequences from the GenBank database were used for comparison. The intraspecific genetic distance within the tick population in this study was 0.019, while the genetic distances between the ticks in this study and those from other countries ranged from 0.009 to 0.031 (Supplementary Table S3).

**Fig. 5:**
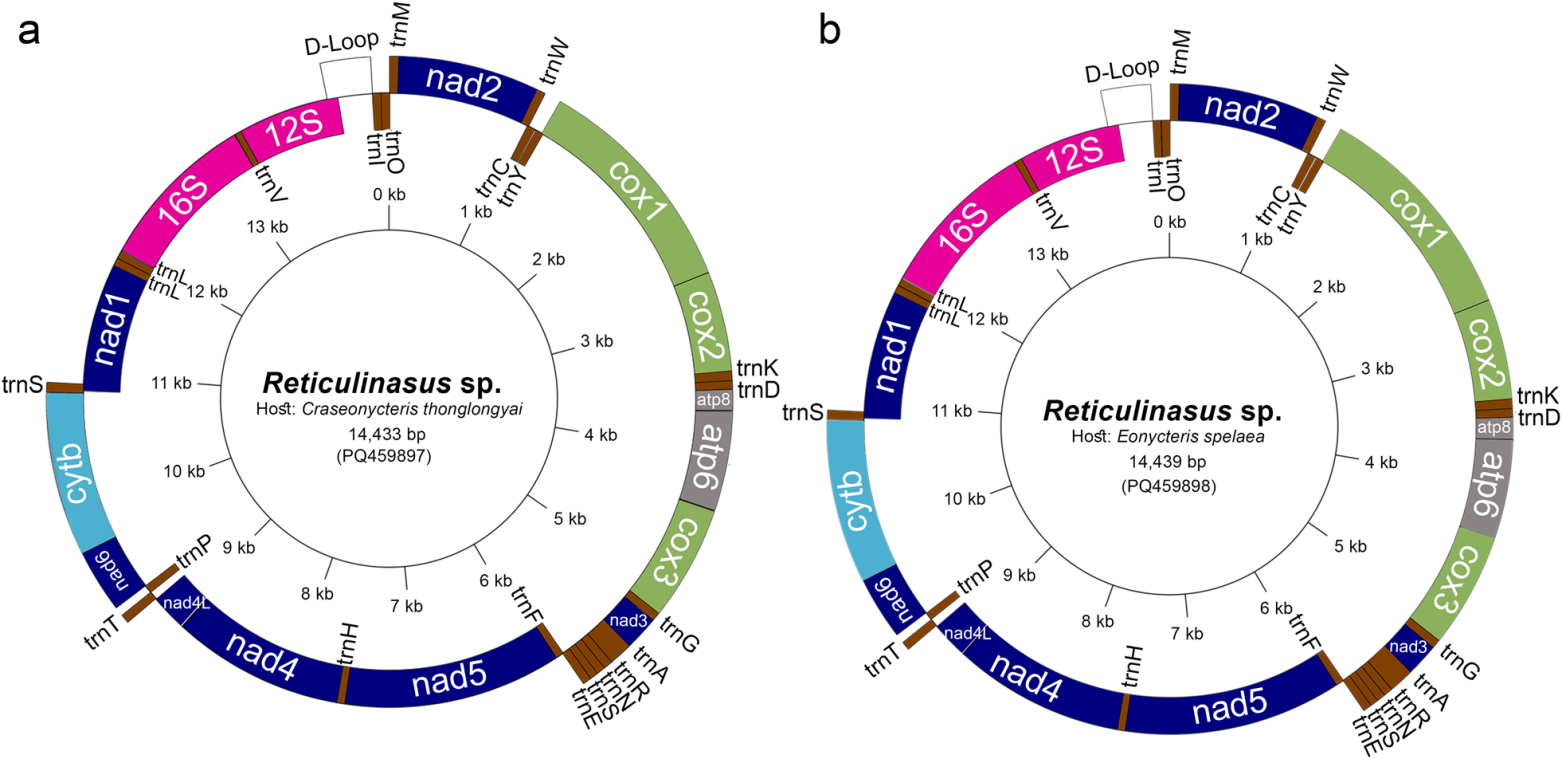
Mitochondrial gene arrangement of *Reticulinasus* sp.: **a** arrangement of genes in a tick collected from *C. thonglongyai*; **b** arrangement of genes in a tick collected from *E. spelaea*. Genes represented on the outer strand correspond to the plus strand (coding strand), while those on the inner strand correspond to the reverse or complementary strand. The colors indicate the following: Green, light blue, and dark blue represent protein-coding genes (PCGs), while pink denotes ribosomal RNA genes, and brown indicates transfer RNA (tRNA) genes

**Fig. 6:**
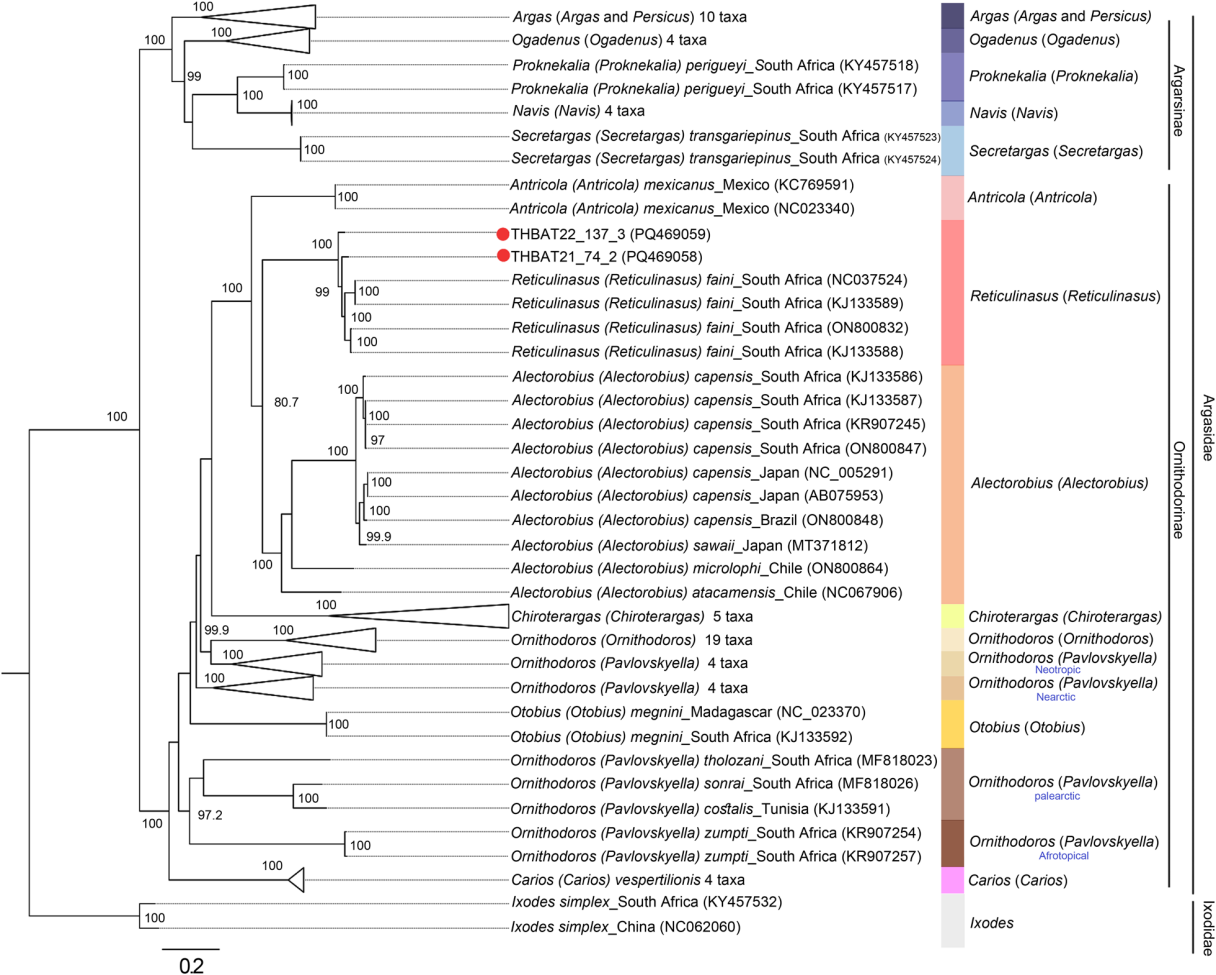
Phylogenetic relationships based on concatenated DNA sequences of 13 protein-coding genes (PCGs) and 2 ribosomal RNA genes. The analysis includes the following PCGs: *nad2, cox1, cox2, atp8, atp6, cox3, nad3, nad5, nad4, nad4L, nad6, cytb*, and *nad1*, totaling 13,445 base pairs (bp). Nodal support is provided for the maximum likelihood analysis, with red dots indicating the tick sequences obtained in this study. Most sequences referenced are from the GenBank database, following the classifications of Mans et al. [5]. The sequences are represented using binomial nomenclature and subgeneric classifications on the basis of Mans et al. [7]. Bootstrap values greater than 80% are indicated

**Fig. 7:**
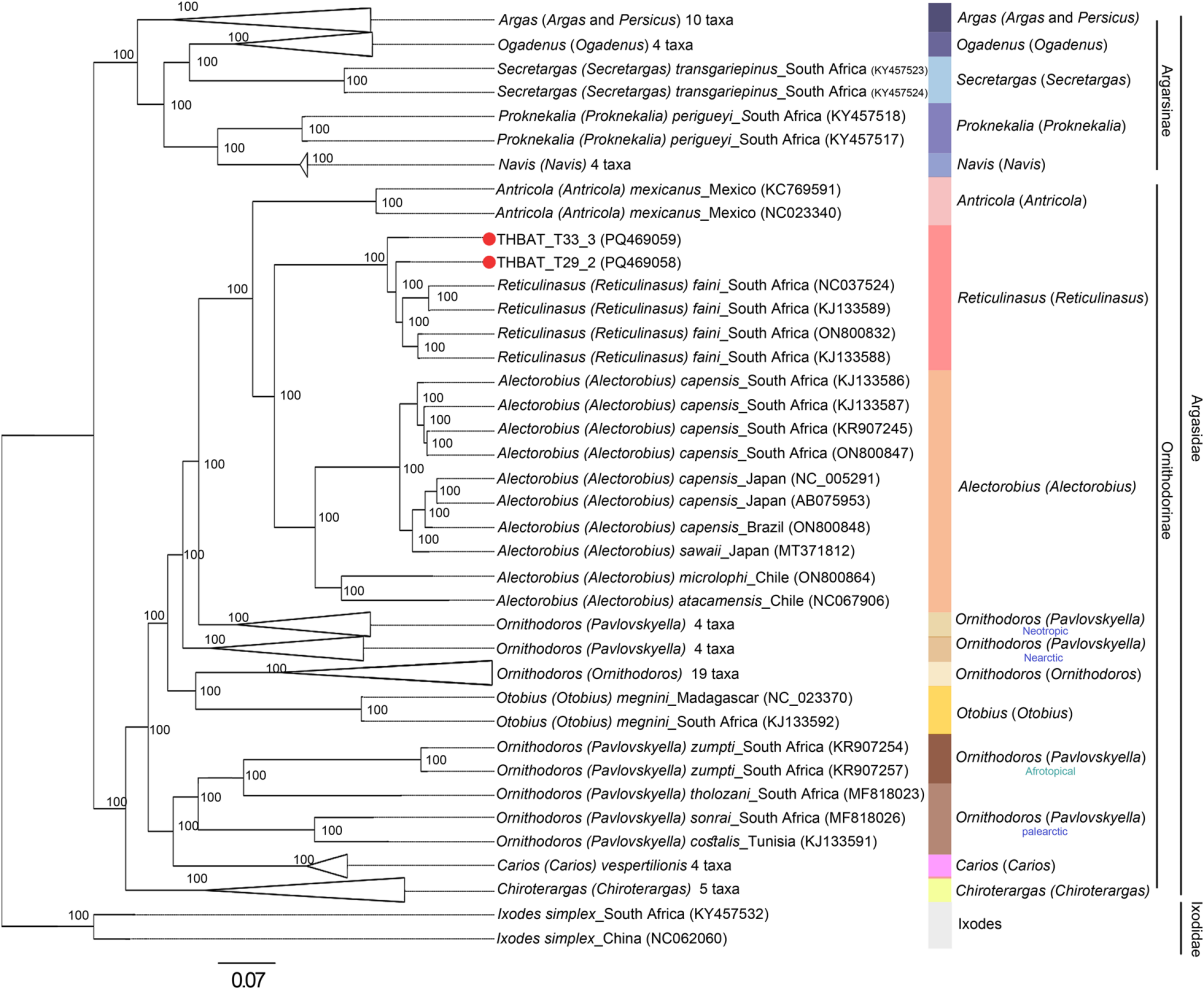
Phylogenetic relationships based on concatenated DNA sequences of 13 protein-coding genes (PCGs) and 2 ribosomal RNA genes. The analysis includes the following PCGs: *nad2, cox1, cox2, atp8, atp6, cox3, nad3, nad5, nad4, nad4L, nad6, cytb*, and *nad1,* comprising a total of 13,445 base pairs (bp). Phylogenetic relationships were inferred using the Bayesian inference method. The red dot denotes the tick sequences identified in this study. Most sequences were obtained from the GenBank database, following the classifications established by Mans et al. [5]. The sequences are presented using binomial nomenclature and subgeneric classifications, as outlined by Mans et al. [7]. Bayesian predictive *P*-values greater than 80% are indicated

The mitochondrial genome sizes of the soft ticks obtained from *C. thonglongyai* and *E. spelaea* bats were 14,433 bp and 14,439 bp, respectively (Fig. 5). The AT content was 75.99% in ticks from *C. thonglongyai* and 76.08% in ticks from *E. spelaea*, while the CG content was 24.01% and 23.92%, respectively. The AT-skew and GC-skew for *Reticulinasus* sp. found on *C. thonglongyai* were 0.068 and −0.363, while those for *Reticulinasus* sp. found on *E. spelaea* were 0.065 and −0.361. Both mitochondrial genomes exhibited identical gene arrangements, comprising 13 protein-coding genes (PCGs), including *nad2*, *cox1*, *cox2*, *atp8*, *atp6*, *cox3*, *nad3*, *nad5*, *nad4*, *nad4L*, *nad6*, *cytb*, and *nad1*, along with 22 transfer RNAs (tRNAs) and 2 ribosomal RNAs (rRNAs), specifically 16S rRNA and 12S rRNA. A total of 9 PCGs and 12 tRNAs were located on the majority strand (J-strand), while the remaining 4 PCGs, 10 tRNAs, and 2 rRNAs were located on the minority strand (N-strand). The start codon for the protein-coding genes was ATN. The gene locations on the mitochondrial map are shown in Fig. 4, with the specific locations and lengths detailed in Table 3. Both maximum likelihood and Bayesian inference analyses of the concatenated sequences of the 13 PCGs and 2 ribosomal genes strongly supported the monophyly of Argasinae and Ornithodorinae. The *Reticulinasus* s.s. clade also displayed clear monophyly (Figs. 6 and 7). However, there were slight differences in the topologies produced by the two methods of analysis. Notably, the same gene arrangement was observed within the genus *Reticulinasus*. Tick samples from this study were placed within the *Reticulinasus* clade and formed taxon branches with nearly 100% bootstrap support, closely clustering with *R. faini* from South Africa.

**Table 3:** Mitochondrion genome organization of ticks in this study

Gene	Strand	*Reticulinasus* sp. from *C. thonglongyai*			*Reticulinasus* sp. from* E. spelaea*		
		Location		Length (bp)	Location		Length (bp)
		Start	Stop		Start	Stop	
*trnM*	+	1	61	61	1	61	61
*nad2*	+	62	1021	960	62	1021	960
*trnW*	+	1020	1080	63	1020	1082	63
*trnC*	−	1073	1133	61	1075	1136	62
*trnY*	−	1144	1203	59	1147	1206	60
*cox1*	+	1196	2734	1539	1199	2737	1539
*cox2*	+	2738	3413	676	2741	3416	676
*trnK*	+	3413	3482	70	3416	3485	70
*trnD*	+	3480	3539	60	3483	3542	60
*atp8*	+	3540	3695	156	3543	3698	156
*atp6*	+	3689	4357	669	3692	4360	669
*cox3*	+	4365	5144	780	4368	5147	780
*trnG*	+	5144	5204	62	5147	5208	62
*nad3*	+	5205	5540	366	5209	5544	366
*trnA*	+	5439	5599	61	5433	5603	61
*trnR*	+	5600	5661	62	5604	5664	61
*trnN*	+	5662	5723	62	5665	5726	62
*trnS*	+	5721	5776	56	5724	5779	56
*trnE*	+	5777	5836	60	5780	5839	60
*trnF*	−	5835	5894	60	5838	5898	61
*nad5*	−	5895	7554	1660	5899	7558	1660
*trnH*	−	7555	7614	60	7559	7617	60
*nad4*	−	7615	8931	1317	7619	8935	1317
*nad4L*	−	8925	9203	279	8929	9207	279
*trnT*	+	9211	9270	60	9215	9274	60
*trnP*	−	9271	9331	61	9275	9335	61
*nad6*	+	9334	9762	429	9338	9766	429
*cytb*	+	9766	10,872	1107	9770	10,876	1107
*trnS*	+	10,879	10,941	63	10,884	10,946	63
*nad1*	−	10,894	11,868	975	10,899	11,873	975
*trnL*	−	11,866	11,926	61	11,871	11,931	61
*trnL*	−	11,927	11,985	59	11,933	11,990	59
*16S*	−	11,994	13,215	2441	11,999	13,221	2441
*trnV*	−	13,220	13,269	59	13,217	13,275	59
12S	−	13,270	14,041	772	13,276	14,049	774
D-loop	+	13,964	14,304	341	13,971	14,309	339
*trnI*	−	14,305	14,371	67	14,310	14,371	62
*trnQ*	−	14,368	14,433	66	14,374	14,439	66

## Discussion

A total of 28 bat species were captured from cave roosts in this study. However, only two species, *C. thonglongyai* and *E. spelaea*, were found to harbor bat ticks. This suggests that, while numerous bat species can host ectoparasites, there may be a host preference for this tick species. *Eonycteris spelaea* inhabits a range of environments, from forests to mixed agricultural areas, and typically roosts near cave entrances, increasing its exposure to the rapidly crawling soft ticks [18]. Previous studies have reported ectoparasites on this bat species in countries such as Singapore and Indonesia [40]. *Carios batuensis* has been found on *E. spelaea* in Peninsular Malaysia [18] and Singapore [41]. However, no previous records of tick infestation in this bat species exist for Thailand, making this the first documentation of tick presence on *E. spelaea* in the country. In this study, only two male *E. spelaea* were captured. Previous research in Singapore suggests that ectoparasites are more commonly found on male *E. spelaea* than on females, as male bats spend more time at roosts due to their role in defending them [42]. *Craseonycteris thonglongyai*, the smallest bat species, is found exclusively in northwest Thailand and southwest Myanmar [43, 44]. To date, no studies have reported tick infestations on *C. thonglongyai*. Therefore, this study marks the first record of tick infestation on *C. thonglongyai*. All the ticks collected in this study were in the larval stage, likely due to the differing feeding behaviors across the life stages of soft ticks. Nymphs and adults typically feed on their hosts for a brief period, ranging from 15 to 60 min, while larvae can feed for several hours to several days [45]. Due to the early developmental stage of the ticks, species identification based solely on morphology is challenging. However, certain morphological characteristics can still be observed in larvae. A previous study in Brazil [23] highlighted some features of immature soft ticks that may assist in species identification. Sonenshine et al. [27] previously described the identification key for tick larvae. Morphological identification reveals that soft ticks within this genus exhibit considerable similarity in appearance. *Reticulinasus faini* closely resembles related species such as *R. salahi*, *R. batuensis*, and *R. piriformis*. Although distinct morphological differences are not prominent, subtle variations exist. For example, *R. salahi* features a larger dorsal plate, *R. batuensis* is distinguished by a piriform Haller’s organ capsule, and *R. piriformis* is characterized by smaller overall dimensions of certain structures. Additionally, *R. faini* can be differentiated from *R. chiropterophila*, found in India, by the position of the hypostome, which arises from the basis capituli. The morphology of the tick larvae analyzed in this study closely resembles *R. faini*. However, their hypostome arises from a median extension, which is not a typical characteristic of *R. faini*. Furthermore, the number of denticles could not be clearly visualized due to damage often incurred during tick removal from the host [46]. Consequently, it is not possible to confirm with certainty that the tick larvae in this study are *R. faini* solely on the basis of morphological examination.

This research represents the first morphological record of tick larvae from Thailand, contributing valuable data for future tick identification efforts. Beyond morphology, molecular analysis offers critical insights for species identification. In this study, 16S rRNA BLASTN and *cox1* BOLD analyses demonstrated a relatively high percentage identity to *R. faini.* This species of tick is commonly found in cracks and crevices near bat roosting sites within caves. The Egyptian fruit bat (*Rousettus aegyptiacus*) has been identified as one of the bat hosts for *R. faini*, with reports of its presence in South Africa and Uganda [47, 48]. *Reticulinasus faini* primarily parasitizes bats and is known to carry various pathogens, including zoonotic agents such as the human-pathogenic Kasokero virus [48], *Rickettsia hoogstraalii*, *Rickettsia lusitaniae* [14], and *Candidatus* Borrelia faini [49]. Of particular concern is the zoonotic pathogen *Candidatus* Borrelia faini, which has been associated with human infections following tick bites from *R. faini* in Africa [49]. This highlights the potential of *R. faini* as a vector for zoonotic pathogens, warranting attention for public health considerations.

This study successfully amplified the complete mitochondrial genome of soft ticks to gain further insights into their characteristics. Analysis of the whole mitochondrial genome revealed minimal differences in genome length between ticks collected from different bat hosts and geographical locations. The AT content was consistent with previous reports of the *R. faini* mitochondrial genome. Previous studies have identified cryptic complexes within the genera *Antricola*, *Nothoaspis*, and *Alectorobius.* The phylogenetic analysis in this study, based on 13 concatenated protein-coding genes (PCGs) and two ribosomal RNA genes from the tick mitochondrial genome, produced a distinct topology compared to the findings of Mans et al. [5], who used five PCGs for their phylogenetic analysis. By including more PCGs, the analysis in this study provides more informative insights. Following the species identification by Mans et al. [5], species should show BLASTN identity values of 96% or higher for 12S rRNA, 16S rRNA, or *cox1* sequences. According to BOLD, the threshold for identity should be 98% or higher [50]. The ticks in this study showed 98.59–99.06% identity with *R. faini* on the basis of partial 16S rRNA sequences using BLASTN results, while they exhibited 94.26–96.09% identity with *R. faini* on the basis of partial *cox1* sequences using BOLD results. The phylogenetic analysis also indicated that the ticks in this study formed distinct branches within the *R. faini* cluster. One limitation of this study is that the ticks analyzed were in the larval stage, which did not display all the characteristic features of *R. faini*, such as the specific position of the hypostome. Additionally, there is a paucity of complete mitochondrial sequences of ticks in public databases, and no reference mitochondrial genome sequences are available for closely related species, including *R. salahi*, *R. madagascariensis*, and *R. chiropterophila*. Until recently, only 16S rRNA and mitochondrial genome sequences of *R. faini* were available in the GenBank database [5, 49, 51]. Notably, *R. faini* was originally described from specimens collected in Congo; however, the genetic sequences currently accessible in public databases do not originate from this type locality. The first genetic sequences attributed to a *Reticulinasus* species were reported by Schuh et al. [51], who identified thousands of nymphs and adult ticks collected from the walls of a cave in Uganda. These specimens were identified as *R. faini* solely on the basis of external morphological characteristics. Subsequent genetic sequences from Zambia [49] and South Africa [5] were also derived from postlarval stages and identified as *R. faini* on the basis of alignment with the sequences reported by Schuh et al. [51]. However, these studies did not include detailed morphological examinations to corroborate the genetic findings. With at least nine recognized species within the genus *Reticulinasus* [8], confidently confirming species identity presents a significant challenge. Closely related species, such as *R. madagascariensis* and *R. chiropterophila*, could also be proposed as tentative diagnoses, especially when considering the geographic distribution of bats that may facilitate the spread of these ticks across continents. The identification of soft ticks primarily relies on the examination of larval stages due to several factors: (a) postlarval stages within a given genus often exhibit significant morphological similarities, (b) postlarval stages of many species remain unidentified, and (c) taxonomic keys for postlarval stages are either limited or nonexistent. This reliance is particularly evident in the genus *Reticulinasus*, which comprises nine recognized species, yet postlarval stages remain unknown for the majority of them. To confirm a new species, both morphological and molecular analyses should be conducted using more extensive reference databases. The nuclear 18-28S ribosomal RNA markers, which are highly conserved within species and genera, may be useful for determining whether the ticks belong to the same genus or closely related genera, or whether they are grouped within a specific subfamily [7]. Despite these limitations, this study presents the first complete mitochondrial genome data for soft ticks associated with bats in Thailand. This tick species represents a largely understudied taxon in contemporary phylogenetic analyses. Its collection from bats, which are protected wildlife species, has posed significant challenges for specimen acquisition, further underscoring the rarity and importance of these findings.

## Conclusions

This study identified tick infestations on two bat species, *C. thonglongyai,* and *E. spelaea*, marking the first report of soft ticks associated with bats in Thailand. The mitochondrial genome sequences exhibited the highest similarity to *R. faini*, a species known to potentially serve as a vector for zoonotic pathogens.

## Supplementary Information

Supplementary Material 1. Table S1. Oligonucleotide primers used in this study for the amplification of short mitochondrial gene fragments. Table S2. Oligonucleotide primers used in this study for the amplification of long mitochondrial gene fragments of *Reticulinasus* ticks. Table S3. Sampling site details and bat captures, including GPS coordinates for each location. Table S4. Frequency of tick infestations on different bat species. Table S5. Intraspecific genetic distances of ticks obtained in this study, compared with reference sequences of the same species from the GenBank database. Table S6. Whole mitochondrial sequences retrieved from the GenBank database.

## Data Availability

The nucleotide sequences obtained in this study were deposited in the GenBank™ database (https://www.ncbi.nlm.nih.gov/nuccore) with the following accession numbers: PQ443920–PQ443932 (cox1), PQ459886–PQ459898 (16S rRNA), and PQ469058–PQ469059 (whole mitochondrial genome).

## Abbreviations

*atp6*: ATP synthase F0 subunit 6
*atp8*: ATP synthase F0 subunit 8
BLASTN: Basic local alignment search tool for nucleotide
BOLD: Barcode of Life Database
*cox1*: Cytochrome oxidase c subunit I
*cox2*: Cytochrome oxidase c subunit II
*cox3*: Cytochrome oxidase c subunit III
*cytb*: Cytochrome b
*nad1*: NADH dehydrogenase subunit 1
*nad2*: NADH dehydrogenase subunit 2
*nad3*: NADH dehydrogenase subunit 3
*nad4*: NADH dehydrogenase subunit 4
*nad4L*: NADH dehydrogenase subunit 4L
*nad5*: NADH dehydrogenase subunit 5
PCR: Polymerase chain reaction
tRNA: Transfer rRNA
