# Supplementary material for: Mitochondrial genome characterization of a Reticulinasus sp. (Argasidae: Ornithodorinae) parasitizing bats in Thailand
Source: Parasit Vectors. 2025 Feb 13;18:52. doi: 10.1186/s13071-025-06697-z (PMC11827295; doi:10.1186/s13071-025-06697-z)
Supplement: Supplementary file 1 — Supplementary Material 1. Table S1. Oligonucleotide primers used in this study for the amplification of short mitochondrial gene fragments. Table S2. Oligonucleotide primers used in this study for the amplification of long mitochondrial gene fragments of Reticulinasus ticks. Table S3. Sampling site details and bat captures, including GPS coordinates for each location. Table S4. Frequency of tick infestations on different bat species. Table S5. Intraspecific genetic distances of ticks obtained in this study, compared with reference sequences of the same species from the GenBank database. Table S6. Whole mitochondrial sequences retrieved from the GenBank database. [file 13071_2025_6697_MOESM1_ESM.docx]

**Table S1.** Oligonucleotide primers used in this study to amplify short mitochondrion gene fragments.

| **Primer set** | **Primer Name** | **Primer sequence (5' to 3')** | **Gene** | **Amplicon size (bp)** | **Reference** |
| --- | --- | --- | --- | --- | --- |
| 1F | Bat_tick_Mt16SF | CCGGTCTCAACTCAGATCAAGT | 16S rRNA | 460 | [29,30] |
| 1R | Bat_tick_Mt16SR | GCTCAATGATTTTTTAAATTGCTG |  |  |  |
| 2F | R_Faini_Cox1_F | GCCATTTTACCGCGATGA | Cytochrome c oxidase subunit 1 *(cox1)* | 845 | [14] |
| 2R | R_Faini_Cox1_R | GGTGGGCTCATACAATAAATC |  |  |  |
| 3F | R_Faini_Cox3_F | GTCGATAAAAGACCTTGACC | Cytochrome c oxidase subunit 3 *(cox3)* | 690 | This study |
| 3R | R_Faini_Cox3_R | TCAGTATCATGCTGCGGC |  |  |  |
| 4F | R_Faini_CytB_F | CCGATCAATCCATGCTAATACAGC | Cytochrome b gene *(cytb)* | 800 | This study |
| 4R | R_Faini_CytB_R | AACCTCTACTGGGCATGCTCC |  |  |  |
| 5F | R_Faini_Mt12S_F | TAAAATTGAGAGCGACGGGCG | 12S rRNA | 530 | This study |
| 5R | R_Faini_Mt12S_R | TCTGGCGAAGCCTGTGCC |  |  |  |

**Note:** R_faini means this primer is designed for *Reticulinasus faini* amplification. F stands for forward primer and R is reverse primer.

**Table S2.** Oligonucleotide primers used in this study to amplify long mitochondrion gene fragments of *Reticulinasus* tick.

| **primer set** | **Primer name** | **Primer sequence (5' to 3')** | **Target of forward primer** | **Target of reverse primer** | **Amplicon  size (bp.)** |
| --- | --- | --- | --- | --- | --- |
| aF | MtG_Cox1Cox3_F | CCTGTATTAGCAGGTGCAATTACC | *cox1* | *cox3* | 2925 |
| aR | MtG_Cox1Cox3_R | ATAGGGGGTCATTGGGTTCC |  |  |  |
| bF | MtG_Cox3Cytb_F | CGCGCTCATCAAACTATAAATATGC | *cox3* | *cytb* | 5475 |
| bR | MtG_Cox3Cytb_R | GGATTTTCAACTGAAAATCCTCCTC |  |  |  |
| cF | MtG_Cytb12S_F | TTCCATTCCACCCATATTTCAC | *cytb* | 12S | 3130 |
| cR | MtG_Cytb12S_R | GGTTAGGACAGTTTGTATATCGC |  |  |  |
| dF | MtG_12SCox1_F | CCTCTAAAAAGCTTAAAAAACCGCC | 12S | *cox1* | 1280 |
| dR | MtG_12SCox1_R | GTTCATCCTGTTCCTGCTCCTC |  |  |  |

**Note:** F stands for forward primer and R is reverse primer

**Table S3.** Details of sampling sites and bat captures, including GPS coordinates for each location.

|  |  |  | **No.** | **Bat species = 28 species** | | | | | | | | | | | | | | | | | | | | | | | |  |  |  |  |
| --- | --- | --- | --- | --- | --- | --- | --- | --- | --- | --- | --- | --- | --- | --- | --- | --- | --- | --- | --- | --- | --- | --- | --- | --- | --- | --- | --- | --- | --- | --- | --- |
| **Province** | **Location** | **GPS coordinate** | **Bats** | ***1*** | ***2*** | ***3*** | ***4*** | ***5*** | ***6*** | ***7*** | ***8*** | ***9*** | ***10*** | ***11*** | ***12*** | ***13*** | ***14*** | ***15*** | ***16*** | ***17*** | ***18*** | ***19*** | ***20*** | ***21*** | ***22*** | ***23*** | ***24*** | ***25*** | ***26*** | ***27*** | ***28*** |
| Nan | Lainan Research and Technology Transfer Station (LRS) | 18°33'14.3'' N100°47'33.2''E | 5 |  | 3 |  |  |  |  |  |  |  |  | 2 |  |  |  |  |  |  |  |  |  |  |  |  |  |  |  |  |  |
| Kanchanaburi | Daowadung Cave (DC) | 14°28'24.4"N 98°50'04.7"E | 62 |  | 1 | 26 |  | 15 |  |  |  |  |  |  | 14 |  |  |  |  |  |  |  |  |  |  |  |  |  |  | 6 |  |
|  | Bat cave (BC) | 14°26'01.2"N 98°51'45.2"E | 18 |  |  | 6 |  | 8 |  |  |  |  | 2 | 1 |  |  |  |  |  |  |  |  |  |  |  |  |  |  |  |  | 1 |
|  | Ma Gleua Cave (MG) | 14°21'31.9"N 98°56'22.8"E | 80 |  |  |  |  |  |  |  |  |  |  | 52 |  |  | 1 |  |  |  |  |  |  | 27 |  |  |  |  |  |  |  |
|  | Dhepnimitra Cave (DH) | 14°24'34.8"N 98°51'50.0"E | 23 |  |  |  |  |  |  |  |  |  |  |  |  | 1 |  |  |  |  |  |  |  |  |  |  | 1 |  |  |  | 21 |
|  | Ta-Klor Cave (TK) | 14°20'34" N 98°57'28"E | 34 |  | 19 |  |  |  |  |  |  |  |  | 1 | 5 |  |  |  |  |  |  |  |  |  |  |  |  |  |  | 9 |  |
|  | Ma Now Phee (MNP) | 14°21'19.6"N 98°56'14.1"E | 64 |  | 9 | 1 |  |  |  |  |  |  |  | 9 |  | 1 |  |  |  |  |  |  |  | 24 |  |  |  |  |  | 2 | 18 |
|  | Phra Cave (PC) | 14°24'36.6"N 98°51'13.5"E | 561 | 140 | 273 |  |  |  | 15 |  |  |  |  | 1 | 3 | 2 | 2 |  | 1 |  |  |  | 1 |  |  |  |  |  |  | 24 | 99 |
|  | Hintok Cutting (HC) | 14°21'52" N 98°56'17"E | 8 |  |  |  |  |  |  |  |  |  |  | 1 | 1 |  |  |  |  |  |  |  |  | 6 |  |  |  |  |  |  |  |
| Ratchaburi | Chomphon Cave (CC) | 13°37'30.3"N 99°35'12.2"E | 17 |  |  | 1 |  | 1 |  | 5 |  |  |  |  | 1 | 2 |  |  |  |  |  |  |  | 7 |  |  |  |  |  |  |  |
|  | Kunchorn Temple (KT) | 13°29'17.0"N 99°42'13.0"E | 5 |  |  | 3 |  |  |  |  |  |  |  |  |  |  |  |  | 1 |  |  |  |  |  |  |  |  |  | 1 |  |  |
|  | Khun Kunchon Cave (KKC) | 13°23'58.1"N 99°45'21.1"E | 4 | 1 |  |  |  | 2 |  |  |  |  |  |  |  | 1 |  |  |  |  |  |  |  |  |  |  |  |  |  |  |  |
| Phetchaburi | Bo Cave (BO) | 13°20'09.1"N 99°45'16.8"E | 4 | 4 |  |  |  |  |  |  |  |  |  |  |  |  |  |  |  |  |  |  |  |  |  |  |  |  |  |  |  |
| Saraburi | Banana Farm (BF) | 14°31'26" N 101°01'36"E | 1 |  |  |  |  |  |  |  |  |  |  |  |  |  |  |  |  |  |  |  |  |  |  |  |  |  | 1 |  |  |
|  | Teak Tree Fores (TT) t | 14°31'33" N 101°01'32"E | 2 |  |  |  |  |  |  |  | 2 |  |  |  |  |  |  |  |  |  |  |  |  |  |  |  |  |  |  |  |  |
|  | Tiger Cave (TC) | 14°31'12.6"N 101°02'13.9"E | 18 |  |  |  |  |  |  |  |  |  |  |  |  |  |  |  |  |  |  |  |  |  |  |  |  |  |  | 18 |  |
|  | Champa Cave (CP) | 14°31'12.6"N 101°02'13.9"E | 3 |  |  |  |  |  |  |  |  |  |  |  |  |  |  |  |  |  |  |  |  |  |  |  |  |  |  | 3 |  |
| Nakorn-sri Thammarat | Khanom Electricity Generating Co Ltd (KEG) | 9°13'48.3"N 99°51'15.6"E | 13 |  |  | 1 | 4 |  |  |  |  |  |  |  |  |  |  | 1 |  |  |  |  |  | 5 | 1 |  |  |  |  | 1 |  |
| Songkla | Saba Yoi (SY) | 6°37′6.0″N 100°57′6.0″E | 7 |  |  | 1 |  |  |  |  |  | 1 |  |  |  |  | 1 | 2 |  |  | 1 |  |  |  |  | 1 |  |  |  |  |  |
| Phang-nga | Yao Noi Island (YN) | 8°6'55.0''N 98°36'34.9''E | 31 |  |  |  | 29 |  |  |  |  |  |  |  |  | 1 |  |  |  |  |  | 1 |  |  |  |  |  |  |  |  |  |
|  | Yao Yai Island (YY) | 8°3'19.1''N 98°35'49.2''E | 2 |  |  |  |  |  |  |  |  |  |  |  |  |  |  |  |  |  |  |  |  |  | 2 |  |  |  |  |  |  |
| Trat | Huang Nam Khiao Waterfall (HN) | 11°39'03.2"N 102°33'36.6"E | 3 |  |  | 3 |  |  |  |  |  |  |  |  |  |  |  |  |  |  |  |  |  |  |  |  |  |  |  |  |  |
|  | Cham's House (CH) | 11°36'14.8"N 102°32'16.9"E | 2 |  |  |  |  |  |  |  |  |  |  |  |  |  |  |  |  |  |  |  |  |  |  |  |  | 2 |  |  |  |
|  | Khlong Chao Waterfall (KC) | 11°38'35.0"N 102°33'15.1"E | 23 |  |  | 23 |  |  |  |  |  |  |  |  |  |  |  |  |  |  |  |  |  |  |  |  |  |  |  |  |  |
|  | Ao Phrao Temple (AP) | 11°35'54.9"N 102°34'00.9"E | 41 |  |  | 39 |  |  |  |  |  |  |  |  |  |  |  |  |  | 1 |  |  |  |  |  |  |  | 1 |  |  |  |
|  |  | **Total** | **1031** |  |  |  |  |  |  |  |  |  |  |  |  |  |  |  |  |  |  |  |  |  |  |  |  |  |  |  |  |

**Note:** Each number corresponds to a bat species: 1: *Taphozous melanopogon,* 2: *Hipposideros gentilis,* 3: *Hipposideros larvatus,* 4: *Hipposideros atrox,* 5: *Hipposideros armiger,* 6: *Hipposideros cineraceus,* 7: *Hipposideros lekaguli,* 8: *Hipposideros diadema,* 9: *Hipposideros bicolor,* 10: *Aselliscus stoliczkanus,* 11: *Rhinolophus coelophyllus,* 12: *Rhinolophus pearsoni,* 13: *Rhinolophus malayanus,* 14: *Rhinolophus stheno*, 15: *Rhinolophus affinis,* 16: *Rhinolophus thomasi,* 17: *Rhinolophus pusillus,* 18: *Rhinolophus acuminatus,* 19: *Rhinolophus refulgens,* 20: *Rhinolophus* sp., 21*: Myotis siligorensis,* 22: *Myotis muricola,* 23: *Kerivoula hardwickii*, 24: *Murina* sp., 25: *Cynopterus brachyotis,* 26: *Eonycteris spelaea,* 27: *Megaderma spasma,* 28: *Craseonycteris thonglongyai*

**Table S4.** The frequency of tick infestation in each species of bats

| **Family** | **Genus** | **Species** | **Number of bats** | **Prevalence (%)** | **Number of ticks** | **Mean intensity** | **Mean abundant** |
| --- | --- | --- | --- | --- | --- | --- | --- |
| Emballonuridae | *Taphozous* | *Taphozous melanopogon* | 145 | - | - | - | - |
| Hipposideridae | *Hipposideros* | *Hipposideros gentilis* | 305 | - | - | - | - |
|  |  | *Hipposideros larvatus* | 104 | - | - | - | - |
|  |  | *Hipposideros atrox* | 33 | - | - | - | - |
|  |  | *Hipposideros armiger* | 26 | - | - | - | - |
|  |  | *Hipposideros cineraceus* | 15 | - | - | - | - |
|  |  | *Hipposideros lekaguli* | 5 | - | - | - | - |
|  |  | *Hipposideros diadema* | 2 | - | - | - | - |
|  |  | *Hipposideros bicolor* | 1 | - | - | - | - |
|  | *Aselliscus* | *Asellicus stoliczlcanus* | 2 | - | - | - | - |
| Rhinolophidae | *Rhinolophus* | *Rhinolophus coelophyllus* | 67 | - | - | - | - |
|  |  | *Rhinolophus pearsonii* | 24 | - | - | - | - |
|  |  | *Rhinolopus malayanus* | 8 | - | - | - | - |
|  |  | *Rhinolophus stheno* | 4 | - | - | - | - |
|  |  | *Rhinolophus affinis* | 3 | - | - | - | - |
|  |  | *Rhinolophus thomasi* | 2 | - | - | - | - |
|  |  | *Rhinolophus pusillus* | 1 | - | - | - | - |
|  |  | *Rhinolophus accumiatus* | 1 | - | - | - | - |
|  |  | *Rhinolophus refulgens* | 1 | - | - | - | - |
|  |  | *Rhinolophus* sp. | 1 | - | - | - | - |
| Vespertilionidae | *Myotis* | *Myotis siligorensis* | 69 | - | - | - | - |
|  |  | *Myotis muricola* | 3 | - | - | - | - |
|  | *Kerivoula* | *Kerivoula hardwickii* | 1 | - | - | - | - |
|  | *Murina* | *Murina* sp. | 1 | - | - | - | - |
| Pteropodidae | *Cynopterus* | *Cynopterus brachyotis* | 3 | - | - | - | - |
|  | *Eonycteris* | *Eonycteris spelaea* | 2 | 1/2 (50%) | 4 | 4 | 2 |
| Craseonycteridae | *Craseonycteris* | *Craseonycteris thonglongyai* | 139 | 33/139 (23.74%) | 92 | 4.788 | 0.661 |
| Megadermatidae | *Megaderma* | *Megaderma spasma* | 63 | - | - | - | - |
|  | | **Total** | 1031 | 34/1,031 (3.30%) |  |  |  |

| **16S** | **(1)** | **(2)** | **(3)** | **(4)** | **(5)** | **(6)** |
| --- | --- | --- | --- | --- | --- | --- |
| **(1) THBAT_T29_2 (PQ459897)** |  |  |  |  |  |  |
| **(2) THBAT_T33_4 (PQ459898)** | 0.019 |  |  |  |  |  |
| **(3) NC037524** | 0.024 | 0.031 |  |  |  |  |
| **(4) ON800832** | 0.014 | 0.019 | 0.024 |  |  |  |
| **(5) KU295470** | 0.017 | 0.022 | 0.026 | 0.002 |  |  |
| **(6) LC634599** | 0.009 | 0.014 | 0.019 | 0.005 | 0.007 |  |

**Table S5.** The intraspecific genetic distances of ticks obtained with in this study compare with other reference sequences in the same species on GenBank database

**Note:** NC037524: *R.faini* from South Africa, ON800832*: R.faini* from South Africa , KU295470: *R.faini* from Uganda, LC634599: *R.faini* from : Lusaka, Zambia.

**Table S6.** Whole mitochondrial sequences retrieved from GenBank database

| **Family** | **Genus** | **species** | **length** | **location** | **GenBank ID** | **Reference** |
| --- | --- | --- | --- | --- | --- | --- |
| Argasidae | *Argas (Argas)* | *Argas* sp. | 14,450 bp | South Africa: Springbok | KC769588 | Burger et al, 2014 |
|  |  | *Argas africolumbae* | 14,440 bp | South Africa | NC_019642 | Mans el al, 2012 |
|  |  | *Argas africolumbae* | 14,439 bp | South Africa: Pretoria | KJ133580 | Mans et al, 2019 |
|  | *Argas (Persicus)* | *Argas miniatus* | 14,416 bp | Brazil: Mato Grosso do Sul, Campo Grande | NC_023371 | Burger et al, 2014 |
|  |  | *Argas walkerae* | 14,437 bp | South Africa: Eastern Cape, Chris Hani District | KJ133584 | Mans et al, 2019 |
|  |  | *Argas walkerae* | 14,437 bp | South Africa: Bushmanland, Nam, Northern Cape | KJ133585 | Mans et al, 2019 |
|  |  | *Argas persicus* | 14,425 bp | Kenya: Machakos | KJ133581 | Mans et al, 2019 |
|  |  | *Argas lagenoplastis* | 14,478 bp | Australia: Northern Territory, Alice Springs | NC023369 | Burger et al, 2014 |
|  |  | *Argas lagenoplastis* | 14,478 bp | Australia: Northern Territory, Alice Springs | KC769587 | Burger et al, 2014 |
|  |  | *Argas japonicus* | 14,479 bp | Japan: Yamanashi | MT371799 | Kelava et at, 2021 |
|  | *Ogadenus (Ogadenus)* | *Ogadenus brumpti* | 14,516 bp | South Africa: Bergpan | KR907229 | Mans et al, 2019 |
|  |  | *Ogadenus brumpti* | 14,516 bp | South Africa: Bergpan | KR907226 | Mans et al, 2019 |
|  |  | *Ogadenus brumpti* | 14,519 bp | South Africa: Lekkersing | KY457510 | Mans et al, 2019 |
|  |  | *Ogadenus brumpti* | 14,519 bp | South Africa: Lekkersing | KY457511 | Mans et al, 2019 |
|  | *Proknekalia (Proknekalia)* | *Proknekalia peringueyi* | 14,584 bp | South Africa: Bloemhof, South Africa | KY457518 | Mans et al, 2019 |
|  |  | *Proknekalia peringueyi* | 14,584 bp | South Africa: Bloemhof, South Africa | KY457517 | Mans et al, 2019 |
|  | *Navus(Navis)* | *Navis striatus* | 14,487 bp | South Africa: Pofadder, Northern Cape | KJ133582 | Mans et al, 2019 |
|  |  | *Navis striatus* | 14,487 bp | South Africa: Pofadder, Northern Cape | KJ133583 | Mans et al, 2019 |
|  |  | *Navis striatus* | 14,472 bp | South Africa: Upington,South Africa | KY457519 | Mans et al, 2019 |
|  |  | *Navis striatus* | 14,476 bp | South Africa: Upington,South Africa | KY457520 | Mans et al, 2019 |
|  | *Secretargas (Secretargas)* | *Secretargas transgariepinus* | 14,534 bp | South Africa | KY457523 | Mans et al, 2019 |
|  |  | *Secretargas transgariepinus* | 14,533 bp | South Africa: Koegas, Northern Cape | KY457524 | Mans et al, 2019 |
|  | *Carios (Anticola)* | *Antricola mexicanus* | 14,415 bp | Mexico: Yucatan | KC769591 | Burger et al, 2014 |
|  |  | *Antricola mexicanus* | 14,415 bp | Mexico: Yucatan | NC023340 | Burger et al, 2014 |
|  | *Carios (Reticulinasus)* | *Reticulinasus faini* | 14,433 bp | South Africa: Kruger National Park, Lanner Gorge Bat Cave | NC037524 | Mans et al,2014 |
|  |  | *Reticulinasus faini* | 14,431 bp | South Africa: Kruger Park | ON800832 | Kneubehl, 2022 |
|  |  | *Reticulinasus faini* | 14,428 bp | South Africa: Kruger National Park, Lanner Gorge Bat Cave | KJ133588 | Mans et al, 2019 |
|  |  | *Reticulinasus faini* | 14,433 bp | South Africa: Kruger National Park, Lanner Gorge Bat Cave | KJ133589 | Mans et al, 2019 |
|  | *Carios (Alectorobius)* | *Alectorobius capensi* | 14,418 bp | South Africa: Cape Town | KJ133586 | Mans et al, 2019 |
|  |  | *Alectorobius capensis* | 14,418 bp | South Africa: St. Croix Island | KJ133587 | Mans et al, 2019 |
|  |  | *Alectorobius capensis* | 14,418 bp | South Africa: St. Croix Island | KR907245 | Mans et al, 2019 |
|  |  | *Alectorobius capensis* | 14,415 bp | South Africa: St. Croix Island | ON800847 | Kneubehl, 2022 |
|  |  | *Alectorobius capensi* | 14,418 bp | Japan | NC_005291 | Fukunaga, 2001 |
|  |  | *Alectorobius capensis* | 14,418 bp | Japan | AB075953 | Fukunaga, 2001 |
|  |  | *Alectorobius capensis* | 14,417 bp | Brazil: Ilha Queimada Grande, Sao Paulo | ON800848 | Kneubehl, 2022 |
|  |  | *Alectorobius sawaii* | 14,417 bp | Japan: Kyoto | MT371812 | Kelava et at, 2021 |
|  |  | *Alectorobius microlophi* | 14,409 bp | Chile: RN Pampa del Tamarugal, Tarapaca | ON800864 | Kneubehl, 2022 |
|  |  | *Alectorobius atacamensis* | 14,430 bp | Chile: Parque Nacional Pan de Azucar | NC067906 | Kneubehl, 2023 |
|  | *Chiropterargas (Chiropterargas)* | *Chiropterargas bouet* | 14,307 bp | South Africa: Prieska | KR907234 | Mans et al, 2019 |
|  |  | *Chiropterargas boueti* | 14,306 bp | South Africa: Prieska | KR907232 | Mans et al, 2019 |
|  |  | *Chiropterargas boueti* | 14,278 bp | South Africa: Bergpan | KR907236 | Mans et al, 2019 |
|  |  | *Chiropterargas boueti* | 14,278 bp | South Africa: Bergpan | KR907239 | Mans et al, 2019 |
|  |  | *Chiropterargas confusus* | 14,287 bp | South Africa: Koegas | KY457514 | Mans et al, 2019 |
|  | *Ornithodoros (Ornithodoros)* | *Ornithodoros kalahariensis* | 14,409 bp | South Africa: Tosca, Northern Cape | KJ133598 | Mans et al, 2019 |
|  |  | *Ornithodoros kalahariensis* | 14,409 bp | South Africa: Droeduin, Northern Cape" | KJ133605 | Mans et al, 2019 |
|  |  | *Ornithodoros pavimentosus* | 14,425 bp | South Africa: Kommegas, Northern Cape | KJ133599 | Mans et al, 2019 |
|  |  | *Ornithodoros noorsveldensis* | 14,420 bp | South Africa: Allemanskraal, Eastern Cape | KJ133601 | Mans et al, 2019 |
|  |  | *Ornithodoros pavimentosus* | 14,425 bp | South Africa: Bushmanland-Nam, Northern Cape | KJ133600 | Mans et al, 2019 |
|  |  | *Ornithodoros savignyi* | 14,401 bp | Sudan: El Obeid | KJ133604 | Mans et al, 2019 |
|  |  | *Ornithodoros savignyi* | 14,401 bp | Sudan: El Obeid | KJ133603 | Mans et al, 2019 |
|  |  | *Ornithodoros porcinus* | 14,379 bp | Kenya: Tsavo West, Ziwani | KJ133595 | Mans et al, 2019 |
|  |  | *Ornithodoros porcinus* | 14,378 bp | Tanzania:Dodoma, Mvumi | AB105451 | Mitani el at, 2004 |
|  |  | *Ornithodoros waterbergensis* | 14,386 bp | South Africa: Bergpan | KR907251 | Mans et al, 2019 |
|  |  | *Ornithodoros waterbergensis* | 14,386 bp | South Africa: Lephalale | KJ133593 | Mans et al, 2019 |
|  |  | *Ornithodoros phacochoerus* | 14,389 bp | South Africa: Pafuri, Kruger National Park | KJ133596 | Mans et al, 2019 |
|  |  | *Ornithodoros phacochoerus* | 14,388 bp | South Africa: Crocodile Bridge, Kruger National Park | KJ133597 | Mans et al, 2019 |
|  |  | *Ornithodoros moubata* | 14,400 bp | South Africa: PaMaenOns, Groot Marico District | KJ133594 | Mans et al, 2019 |
|  |  | *Ornithodoros moubata* | 14,398 bp | Tanzania | AB073679 | Fukunaga, et al, 2001 |
|  |  | *Ornithodoros moubata* | 14,398 bp | Tanzania | NC004357 | Fukunaga, et al, 2001 |
|  |  | *Ornithodoros compactus* | 14,400 bp | South Africa: Voelklip, Northern Cape | KJ133590 | Mans et al, 2019 |
|  |  | *Ornithodoros compactus* | 14,400 bp | South Africa: Klein Noute | KY457534 | Mans et al, 2019 |
|  |  | *Ornithodoros compactus* | 14,400 bp | South Africa: Klein Noute | KY457533 | Mans et al, 2019 |
|  | *Ornithodoros (Pavloskyella)* | *Pavloskyella brasiliensis* | 14,489 bp | Brazil: Rio Grande do Sul, Sao Francisco de Paula | NC023373 | Burger et al, 2014 |
|  |  | *Pavloskyella brasiliensis* | 14,489 bp | Brazil: Rio Grande do Sul, Sao Francisco de Paula | KC769593 | Burger et al, 2014 |
|  |  | *Pavloskyella rostratus* | 14,452 bp | Brazil: Mato Grosso do Sul, Corumba | NC023372 | Burger et al, 2014 |
|  |  | *Pavloskyella rostratus* | 14,452 bp | Brazil: Mato Grosso do Sul, Corumba | KC769592 | Burger et al, 2014 |
|  |  | *Pavloskyella turicata* | 14,458 bp | South Africa | MF818021 | Mans et al, 2019 |
|  |  | *Pavloskyella turicata* | 14,467 bp | South Africa | MG593162 | Mans et al, 2019 |
|  |  | *Pavloskyella parkeri* | 14,437 bp | South Africa | MF818029 | Mans et al, 2019 |
|  |  | *Pavloskyella tholozani* | 14,407 bp | South Africa | MF818023 | Mans et al, 2019 |
|  |  | *Ornithodoros hermsi* | 14,430 bp | South Africa | MF818032 | Mans et al, 2019 |
|  |  | *Ornithodoros sonrai* | 14,430 bp | South Africa | MF818026 | Mans et al, 2019 |
|  |  | *Ornithodoros costalis* | 14,442 bp | Tunisia: Outhna | KJ133591 | Mans et al, 2019 |
|  |  | *Ornithodoros zumpti* | 14,434 bp | South Africa: Grahamstown | KR907254 | Mans et al, 2019 |
|  |  | *Ornithodoros zumpti* | 14,438 bp | South Africa: Cathcart | KR907257 | Mans et al, 2019 |
|  | *Otobius (Otobius)* | *Otobius megnini* | 14,430 bp | Madagascar: Tana | NC_023370 | Burger et al, 2014 |
|  |  | *Otobius megnini* | 14,426 bp | South Africa: Port Elizabeth, Eastern Cape | KJ133592 | Mans et al, 2019 |
|  | *Argas (Carios)* | *Carios vespertilionis* | 14,521 bp | Spain: Ciutadella, Menorca | MT680027 | Mans et al, 2021 |
|  |  | *Carios vespertilionis* | 14,520 bp | Spain: Ciutadella, Menorca | MT680028 | Mans et al, 2021 |
|  |  | *Carios vespertilionis* | 14,525 bp | Japan: Nagano | MT762370 | Mans et al, 2021 |
|  |  | *Carios vespertilionis* | 14,521 bp | Spain: Ciutadella, Menorca | NC060373 | Mans et al, 2021 |
| Ixodidae | *Ixodes* | *Ixodes simplex* | 14,551 bp | South Africa: Bakwena Cave | KY457532 | Mans et al, 2019 |
|  |  | *Ixodes simplex* | 14,556 bp | China | NC062060 | Tian et al, 2022 |
